# θ-Band Cortical Tracking of the Speech Envelope Shows the Linear Phase Property

**DOI:** 10.1523/ENEURO.0058-21.2021

**Published:** 2021-08-24

**Authors:** Jiajie Zou, Chuan Xu, Cheng Luo, Peiqing Jin, Jiaxin Gao, Jingqi Li, Jian Gao, Nai Ding, Benyan Luo

**Affiliations:** 1 Key Laboratory for Biomedical Engineering of Ministry of Education, College of Biomedical Engineering and Instrument Sciences, Zhejiang University, Hangzhou 310027, China; 2 Research Center for Advanced Artificial Intelligence Theory, Zhejiang Lab, Hangzhou 311121, China; 3 Department of Neurology, First Affiliated Hospital, School of Medicine, Zhejiang University, Hangzhou 310003, China; 4 Department of Rehabilitation, Hangzhou Mingzhou Brain Rehabilitation Hospital, Hangzhou 311215, China

**Keywords:** neural entrainment, phase resetting, EEG

## Abstract

When listening to speech, low-frequency cortical activity tracks the speech envelope. It remains controversial, however, whether such envelope-tracking neural activity reflects entrainment of neural oscillations or superposition of transient responses evoked by sound features. Recently, it is suggested that the phase of envelope-tracking activity can potentially distinguish entrained oscillations and evoked responses. Here, we analyze the phase of envelope-tracking in humans during passive listening, and observe that the phase lag between cortical activity and speech envelope tends to change linearly across frequency in the θ band (4–8 Hz), suggesting that the θ-band envelope-tracking activity can be readily modeled by evoked responses.

## Significance Statement

During speech listening, cortical activity tracks the speech envelope, which is a critical cue for speech recognition. It is debated, however, what is the neural mechanism generating the envelope-tracking responses. Previous work has shown that δ-band envelope tracking responses recorded during music listening cannot be explained by a simple linear-system model. Here, however, we demonstrate that θ-band envelope tracking responses recorded during speech listening shows the linear phase property, which can be well explained by a linear-system model.

## Introduction

The speech envelope, i.e., temporal modulations below 20 Hz, is critical for speech recognition (Drullman et al., 1994; Shannon et al., 1995; Shamma, 2001; Elliott and Theunissen, 2009; Ding et al., 2017), and large-scale cortical activity measured by MEG and EEG can track the speech envelope (Lalor et al., 2009; Ding and Simon, 2012b; Wang et al., 2012; Peelle et al., 2013; Doelling et al., 2014; Harding et al., 2019). Since the slow temporal modulations in speech are highly related to the ∼5-Hz syllabic rhythm in speech, it has been hypothesized that θ-band neural synchronization to temporal modulations in speech provides a plausible mechanism to segment continuous speech into the perceptual units of syllables (Giraud and Poeppel, 2012; Poeppel and Assaneo, 2020).

Although low-frequency neural synchronization to slow temporal modulations has been extensively studied and is hypothesized to play a critical role in auditory perception, there is considerable debate about how it is generated (Ding and Simon, 2013; Doelling et al., 2014; Ding et al., 2016b; Haegens and Zion Golumbic, 2018; Zoefel et al., 2018; Alexandrou et al., 2020). On the one hand, it has been hypothesized that the low-frequency neural response to speech is generated by resetting the phase of intrinsic neural oscillations (Kayser et al., 2008; Lakatos et al., 2008, 2009; Schroeder et al., 2008; Kayser, 2009). On the other hand, it has been hypothesized that it is a sequence of transient responses evoked by sound features in speech (Lalor et al., 2009; Ding and Simon, 2012a,b). Distinguishing these two hypotheses, however, turns out to be extremely hard. For example, early studies have shown that the phase but not power of θ-band cortical activity is synchronized to speech (Luo and Poeppel, 2007; Howard and Poeppel, 2010), which supports the phase resetting hypothesis. It has been argued, however, that the same phenomenon can be observed for evoked responses, attributable to the different statistical sensitivity of response phase and power (Ding and Simon, 2013; Shah et al., 2004; Yeung et al., 2004). Furthermore, later studies observe consistent power and phase changes in the θ band during speech listening (Howard and Poeppel, 2012).

The phase resetting hypothesis and the evoked response hypothesis motivate different computational models for the neural responses to speech. Based on the evoked response hypothesis, the speech response can be simulated based on a linear time-invariant system, in which the phase lag between stimulus and response dramatically varies across frequency. A recent study, however, shows that neural synchronization to music violates the phase lag property predicted by the evoked response model, when listeners perform a pitch judgment task (Doelling et al., 2019). Instead, the response phase is more consistent with the prediction of a nonlinear oscillator model. This result suggests that cortical synchronization to music is potentially generated by more complicated nonlinear mechanisms than superposition of evoked responses. Since the evoked response model and the nonlinear oscillator model in Doelling et al. (2019) are computationally explicit, here we focus on these two models and test which model can better describe the neural response to speech.

The study by Doelling et al. (2019) questions the validity of using evoked response models to analyze neural activity synchronized to sound rhythms, since such models fail to predict the neural response phase during music listening. It remains possible, however, that the neural encoding scheme depends on the properties of sound. For example, the nonlinear oscillator models may be more appropriate for music, which is highly rhythmic, while the evoked response models may be sufficient to model the response to less rhythmic sound such as speech. It is also possible that the neural encoding scheme depends on the modulation frequency and appears to be different for music, which contains strong temporal modulations below 2 Hz, and speech, which contains strong temporal modulations around 5 Hz (Ding et al., 2017). Finally, it is also possible that active listening engages phase resetting mechanisms more than passive listening. Therefore, the primary goal of the current study is to quantify the phase lag property of the cortical response to speech during passive listening, and test whether it is more consistent with the prediction of the evoked response model or the nonlinear oscillator model in Doelling et al. (2019).

## Materials and Methods

### Participants

This study involved 15 healthy individuals (five males; 54.6 ± 10.12 years), who were right-handed with no history of neurologic diseases. Written informed consent was provided by participants.

### Stimuli

Natural speech included two chapters from a novel, *The Supernova Era* by Cixin Liu (chapter 16, Fun country, and chapter 18, Sweet dream period). The story was narrated in Mandarin by a female speaker and digitized at 48 kHz. The speech was clear and highly intelligible. The two chapters were 34 min and 25 min in duration, respectively. Recordings of the two chapters were concatenated.

### Procedures

All participants listened to speech while EEG responses were recorded. Speech was presented binaurally through headphones at a comfortable sound level. The experiment was separated into 2 d. On each day of the experiment, the spoken narrative was presented once. The 59-min speech stimulus was presented twice and therefore the total speech stimulus was almost 2 h, which was longer than the stimulus duration in most studies. The purpose of having the long stimulus was to reliably estimate the response phase. No other tasks were given, and therefore the participants listened passively.

### EEG recording and signal preprocessing

EEG signals were recorded using a 64-electrodes BrainCap (Brain Products GmbH) in the international 10–20 system, and one of the 64 electrodes was placed under the right eye to record electrooculogram (EOG). EEG signals were referenced online to FCz, but were referenced offline to a common average reference (Poulsen et al., 2007). The EEG signals were filtered online with a 50-Hz notch filter to remove line noise (12th order zero-phase Butterworth filter), a low-pass antialiasing filter (70-Hz cutoff, eighth order zero-phase Butterworth filter), and a high-pass filter to prevent slow drifts (0.3-Hz cutoff, eighth order zero-phase Butterworth filter). The signals were sampled at 1 kHz. The EEG signal was processed following the procedure in Zou et al. (2019). All preprocessing and analysis in this study were conducted in the MATLAB software (The MathWorks).

EEG recordings were low-pass filtered below 50 Hz with a zero-phase anti-aliasing FIR filter (implemented using a 200-ms Kaiser window) and down-sampled to 100 Hz. EOG artifacts were regressed out based on the least-squares method. Similar to previous studies (Ding and Simon, 2012a,b), the speech response was averaged over the two representations on two recording days to increase the signal-to-noise ratio.

The envelopes of stimuli reflected how sound intensity fluctuated over time and were extracted by applying full-wave rectification to the stimulus. Similar to the preprocessing of EEG recordings, the envelopes were further low-pass filtered below 50 Hz with a zero-phase anti-aliasing FIR filter (implemented using a 200-ms Kaiser window) and down-sampled to 100 Hz.

### Phase coherence analysis

To characterize the stimulus-response phase lag, the stimulus and response were both transformed into the frequency domain. Specifically, the acoustic envelope and EEG response were segmented into non-overlapping 2-s time bins, and all segments were converted into the frequency domain using the fast Fourier transform (FFT) algorithm. The response phase and stimulus phase were denoted as *α_ft_* and *β_ft_*_,_ respectively, for frequency bin *f* and time bin *t*, and the stimulus-response phase lag was calculated as *θ_ft_* = *α_ft_* − *β_ft_*. The coherence of the phase lag across time bins, also known as the cerebro-acoustic phase coherence (Peelle et al., 2013), was calculated using the following equation:

$$C(f)=\frac{{\left(\sum_{t=1}^{T}cos(\theta_{ft})\right)}^{2}+{\left(\sum_{t=1}^{T}sin(\theta_{ft})\right)}^{2}}{T},$$

where *C*(*f*) was the phase coherence in frequency bin *f*, and *T* is the total number of time bins. The phase coherence was independently calculated for each electrode and then averaged using the arithmetic mean. The phase coherence is in the range of 0–1, and higher phase coherence indicated that the response phase was more precisely synchronized to the stimulus phase.

In the response topography analysis, we considered a signed phase coherence. Specifically, we chose channel Fz as a reference. For each electrode, if the phase difference between this electrode and electrode Fz was larger than 90°, the phase coherence was negated. Otherwise, the phase coherence was kept positive. The signed phase coherence could illustrate the phase relationship between electrodes on top of showing the phase coherence. Since the phase coherence was strongest in central-frontal electrodes, fourteen centro-frontal electrodes, i.e., Fz, F1, F2, F3, F4, FC1, FC2, FC3, FC4, Cz, C1, C2, C3, and C4, were used to characterize the phase-frequency relationship.

### Phase-frequency relationship

The stimulus-response phase lag at frequency *f*, i.e., *θ_f_*, was calculated by averaging *θ_ft_* over electrodes and all 2-s time bins using the circular average (Fisher, 1993). The group delay is defined based on the first-order derivative of the stimulus-response phase lag over frequency, i.e., *d*(*f*) = (*θ*(*f*) – *θ*(*f* + Δ*f*))/2πΔ*f*, which reflects how quickly a change in the stimulus is reflected in the response (Oppenheim et al., 1997). To calculate the group delay, we unwrapped the phase lag, fitted the phase lag with a straight line, and divided the slope of the straight line by 2π.

To evaluate the linearity of the phase-frequency curve, we defined a linearity measure as *L*(*f*)* *=* *1/*|θ*(*f*) + *θ*(*f +* 2Δ*f*)* *−* *2*θ*(*f* + Δ*f*)|. This measure was the reciprocal of the absolute value of the second-order derivative of the phase-frequency curve and different electrodes was pooled with averaging. If phase lag linearly changed with frequency, the second-order derivative was 0 and the linearity measure was positive infinity. A large *d*_2_(*f*) indicated a roughly linear phase-frequency curve.

Since the phase-frequency curve was approximately linear in the θ band, we fitted the actual phase-frequency curve in this frequency range using a linear function: *θ_L_*(*f*) = *kf* + *b*, 4 ≤ *f *<* *8. The slope parameter *k* and the intercept parameter *b* were fitted using the least-squares method, and the slope parameter *k* denoted the mean group delay between 4 and 8 Hz.

### Statistics

#### Phase coherence

To evaluate whether the phase coherence at a frequency was significantly higher than chance, we estimated the chance-level phase coherence with a permutation strategy (Peelle et al., 2013; Harding et al., 2019). After the speech envelope and EEG response were segmented into 2-s time bins, we shuffled all time bins for the speech envelope so that the envelope and response were randomly paired. We calculated the phase coherence for the phase lag between the EEG response and randomly paired speech envelope. This procedure was repeated 5000 times, creating 5000 chance-level phase coherence. We averaged the phase coherence value over electrodes and participants, for both the actual phase coherence and the 5000 chance-level phase coherence. The significance level of the phase coherence at a frequency was (*N *+* *1)/5001, if it was lower than *N* out of the 5000 chance-level coherence at that frequency (one-sided comparison).

#### Linearity

The chance-level linearity measure of the phase-frequency curve was estimated using a similar procedure. The linearity measure was significantly larger than chance with the significance level being (*N *+* *1)/5001, if it was smaller than *N* of the 5000 chance-level values (one-sided comparison).

For the comparisons of the linearity measure of different frequency bands, statistical tests were performed using bias-corrected and accelerated bootstrap (Efron and Tibshirani, 1994). In the bootstrap procedure, the differences between two frequency bands were resampled with replacement 5000 times. Each time the data sampled were averaged across participants, therefore a total of 5000 mean values were produced. If *N* out of the 5000 mean values were greater (or smaller) than 0, the significance level was 2(*N *+* *1)/5001 (two-sided comparison).

#### Correlation between phase coherence and phase linearity

For significance test of correlation between phase coherence and phase linearity, we used two-tailed Student’s *t* test. When multiple comparisons were performed, the *p* value was further adjusted using the false discovery rate (FDR) correction (Amini and Hochberg, 1995).

### Model simulation

**Figure 1.**
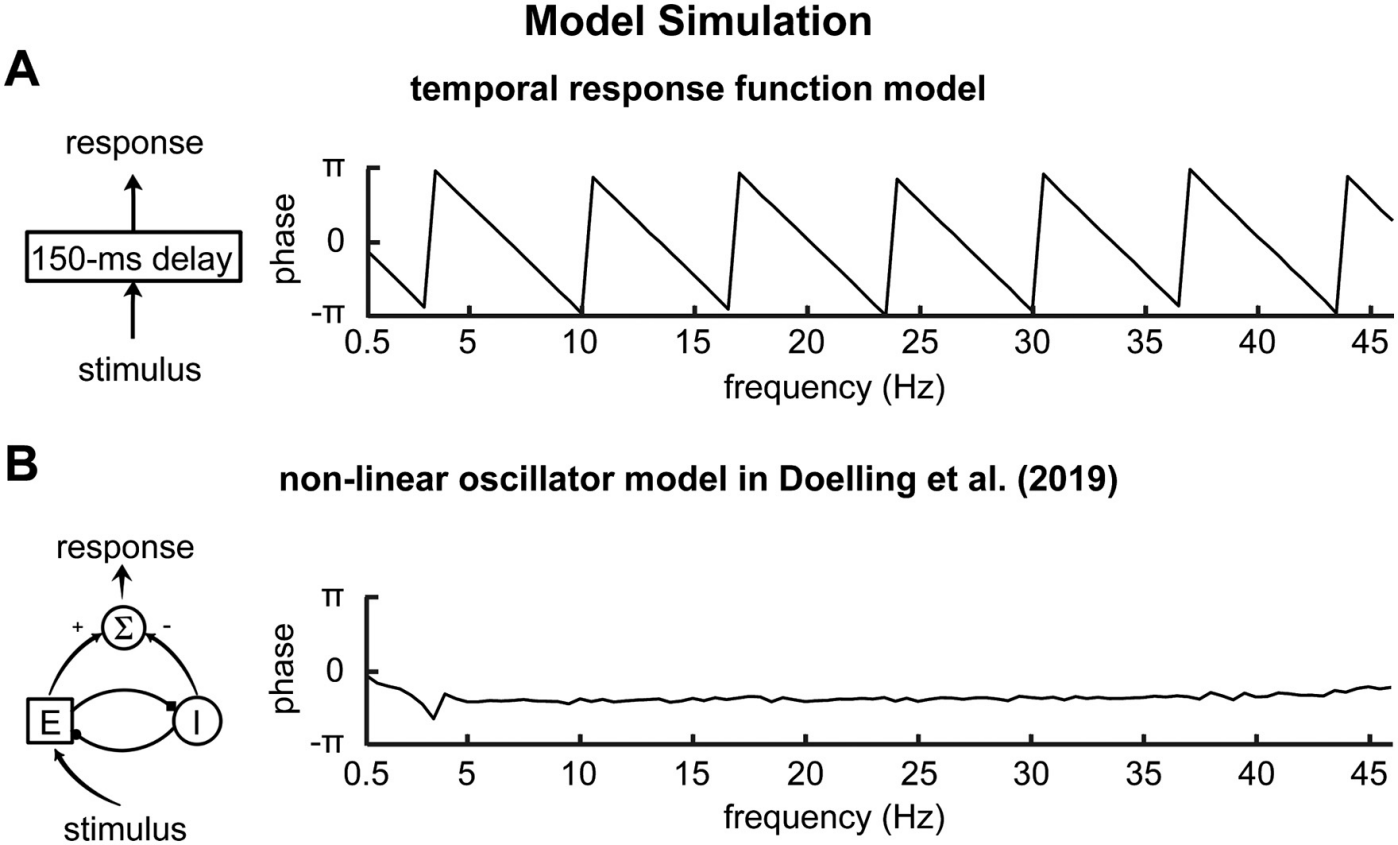
Simulated phase-frequency curve. The curve shows the stimulus-response phase lag as a function of response frequency. Panels ***A***, ***B*** separately show the results simulated based on the evoked response model and the nonlinear oscillator model proposed in Doelling et al. (2019). These two models are based on the evoked response hypothesis and the phase resetting hypothesis, respectively. In the current evoked response model, the response evoked by a unit change in stimulus is a delayed impulse and the function of the model is to delay the stimulus by 150 ms. Such a model predicts that the stimulus-response phase lag changes linearly across frequency. Consequently, the phase-frequency curve appears to have a sawtooth shape. The nonlinear oscillator model, in contrast, predicts that the stimulus-response phase lag only changes in a very limited range across frequency.

We simulated the neural response to speech using two models. One was the evoked response model, in which the simulated neural response was simply the speech envelope convolving the response evoked by a unit change in the speech envelope. This model was formulated as follows:

$$r\left(t\right)=\overset{T}{\underset{0}{\int }}h(\tau )A\left(t-\tau \right)d\tau ,$$

where *r*(*t*) and *A*(*t*) were the simulated neural response and the speech envelope, respectively. The *h*(*t*) described the neural response evoked by a unit change in the stimulus. In the illustration in Figure 1*A*, *h*(*t*) was a unit impulse function with 150-ms response latency, i.e., *h*(*t*) = *δ*(*t *−* *150 ms), and in this case *r*(*t*) = *A*(*t *−* *150 ms). The simulation results would not be affected as long as *h*(*t*) had a symmetric waveform centered at 150 ms.

The other model was the nonlinear oscillator model proposed by Doelling et al. (2019). The oscillator model was formulated as follows:

$$\tau \frac{dI(t)}{dt}=-I(t)+S(\rho_{I}+bE(t)-dI(t)),$$

where *A*(*t*) was the speech envelope. *E*(*t*) and *I*(*t*) simulated the responses from an excitatory and an inhibitory neural population, respectively. The output of this model was the difference between excitatory and inhibitory populations, i.e., *E*(*t*) − *I*(*t*). *S* denoted the sigmoid function. All the parameters were the same as in Doelling et al. (2019), i.e., *a *=* b* = *c *=* *10, *d* = −2, *ρ_E_* = 2.3, *ρ_I_* = −3.2, *κ* = 1.5, and *τ* = 66 ms. Interpretations of the parameters could be found in Doelling et al. (2019). The model was simulated using the ode3 method in MATLAB Simulink (The MathWorks) and the time step was 1 ms.

In the simulations, the input was the envelope of the entire 59-min speech stimulus, and the input-output phase lag was calculated the same way it was calculated in the EEG analysis.

## Results

**Figure 2.**
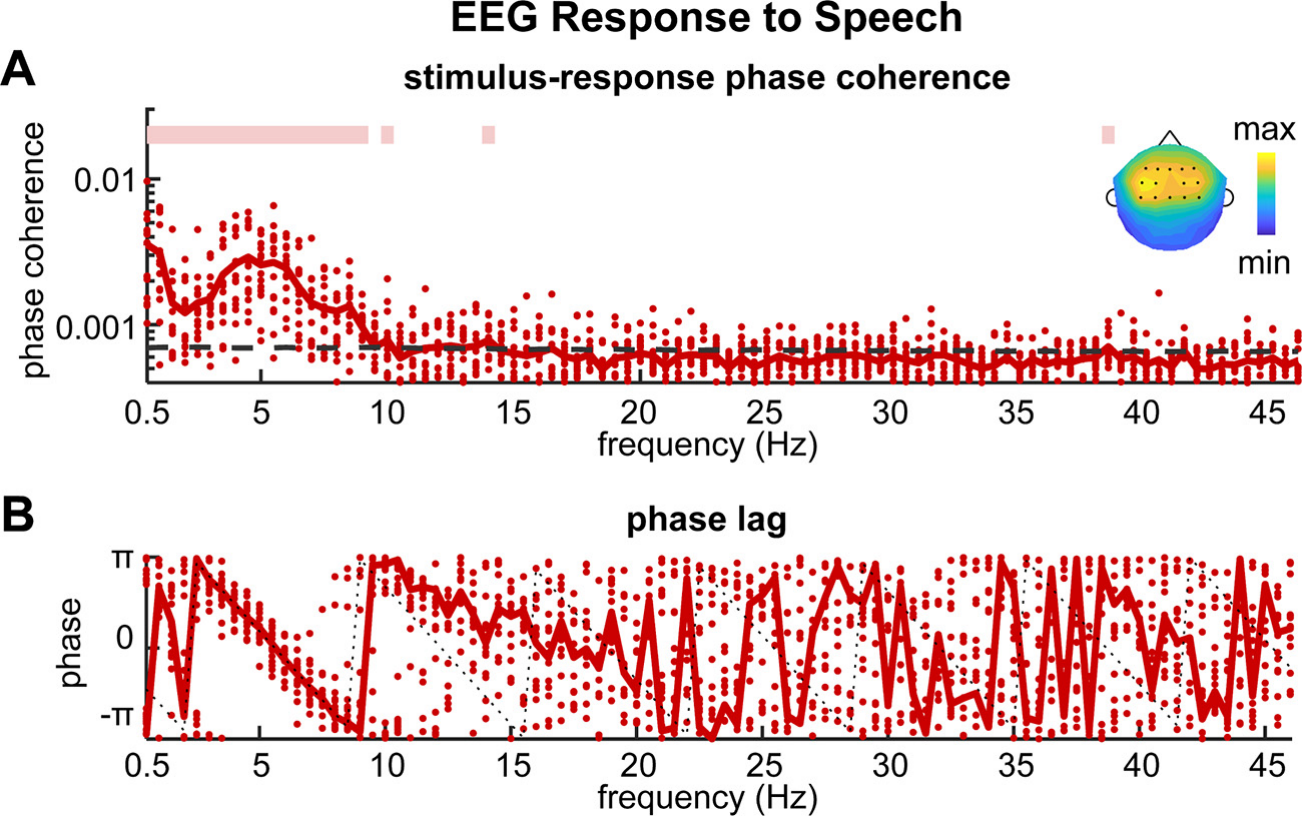
Phase analysis of the envelope-tracking response. ***A***, The phase coherence spectrum shows how precisely the response phase is phase locked to the stimulus. The dashed black line shows the 99% confidence interval of the chance-level phase coherence. The pink line on top denotes the frequency bins in which phase coherence is significantly higher than chance (*p *<* *0.01, permutation test, FDR corrected). The topography shows the signed phase coherence averaged between 0.5 and 9 Hz. The dark dots denote the 14 centro-frontal electrodes selected for the further phase analysis. ***B***, The phase-frequency curve. The phase lag appears to linearly decrease over frequency in the frequency band where the phase coherence was higher than chance. The black dotted lines are fitted based on the phase lag in the θ band. Each red dot denotes a participant.

We first analyzed in which frequency bands and EEG electrodes reliable cortical synchronization to speech was observed. The coherence of the stimulus-response phase lag was separately calculated in each frequency bin. Significant phase coherence was most reliably observed below 9 Hz. The topography of the low-frequency neural responses (<9 Hz) showed a centro-frontal distribution (Fig. 2*A*, upper right corner).

**Figure 3.**
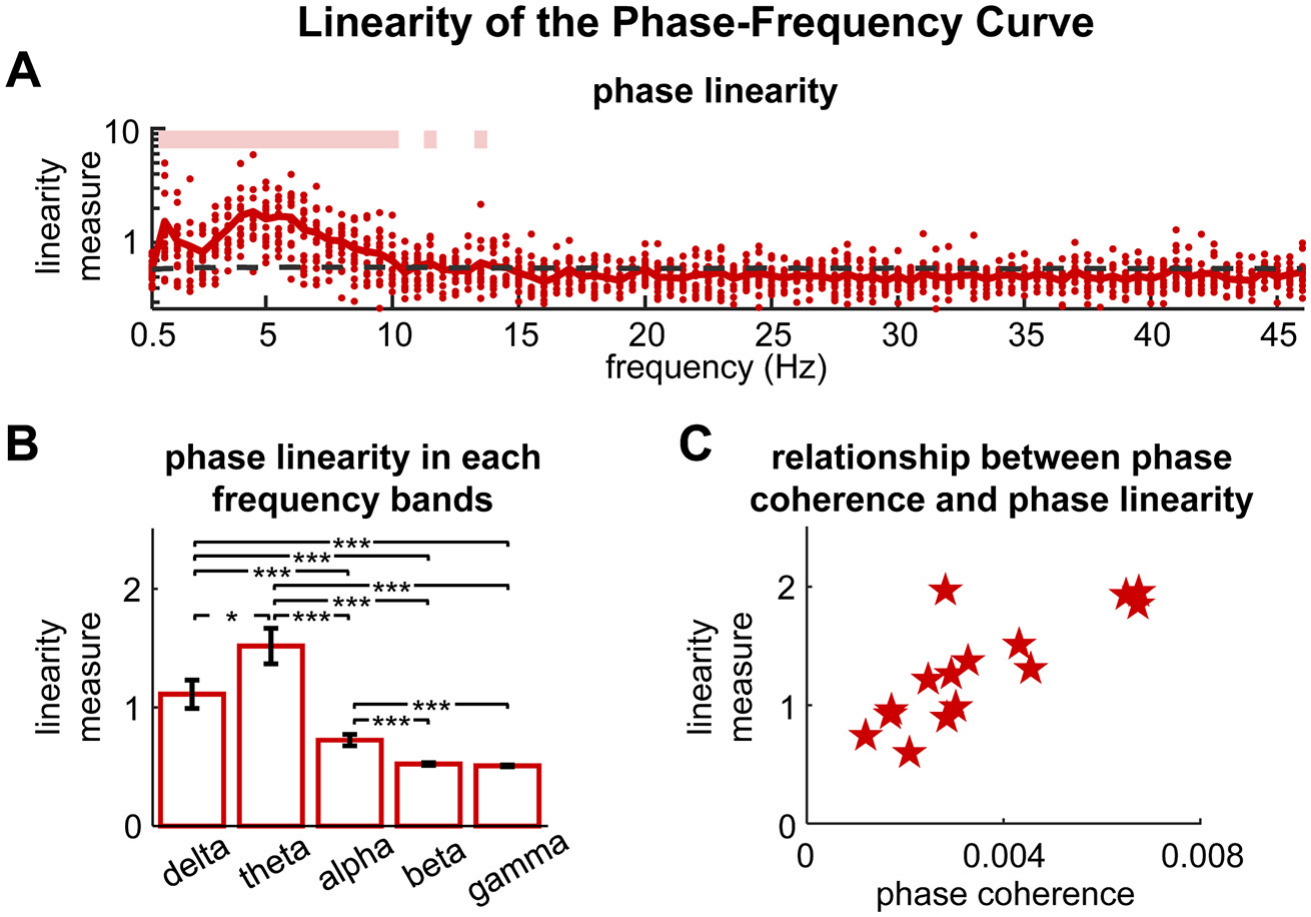
Linearity of the phase-frequency curve. ***A***, Phase linearity as a function of frequency. The dashed black line shows the 99% confidence interval of the chance-level phase linearity. The pink lines on top denotes the frequency bins in which the phase linearity is significantly higher than chance (*p *<* *0.01, permutation test, FDR corrected). Each red dot denotes a participant. ***B***, The comparison of phase linearity across frequency bands. Error bars represent 1 standard error of the mean across participants. Significant differences between frequency bands are indicated by stars; *p <* *0.05, ***p <* *0.001 (bootstrap, FDR corrected). ***C***, The relationship between phase coherence and phase linearity. The phase coherence and the phase linearity are both averaged within the θ band. Each red marker denotes a participant. Participants with higher phase coherence generally show better linearity (*R *=* *0.805, *p *=* *3 × 10^−4^, two-tailed Student’s *t* test).

We next analyzed how the stimulus-response phase lag varied across frequency. The phase lag appeared to change linearly over frequency in the frequency range where the phase coherence was higher than chance (Fig. 2*B*). We then evaluated the linearity of the phase-frequency curve (see Materials and Methods). As shown in Figure 3*A*, the linearity measure was significantly higher than chance in the low-frequency bands (*p *<* *0.01, permutation test, FDR corrected) and peaked in the θ band. We compared the averaged phase linearity across δ (1–4 Hz), θ, α (8–13 Hz), β (13–30 Hz), and γ (30–45 Hz) bands. As shown in Figure 3*B*, the phase linearity was significantly higher in the θ band than other frequency bands (θ band vs δ band: *p *=* *0.043; θ band vs α, β, or γ band: *p *=* *4 × 10^−4^, bootstrap, FDR corrected). In order to estimate the group delay in the θ band, we used a straight line to fit the linear trend, which was shown by the dotted gray line in Figure 2*B*. The mean group delay in the θ band, i.e., slope of the linear fit in Figure 2*B*, was 156 ± 50 ms.

Additionally, we investigated whether the phase-frequency curve tended to be more linear for participants who showed higher phase coherence. As the θ band shows significant and highest phase linearity (Fig. 3*A*,*B*), we compared the averaged phase linearity and the average phase coherence in the θ band (Fig. 3*C*). The phase linearity and the phase coherence were significantly correlated at the individual level (*R* = 0.805, *p *=* *3 × 10^−4^, two-tailed Student’s *t* test).

## Discussion

The current study investigates the phase property of the EEG response to speech during passive listening. It is shown that the stimulus-response phase lag is approximately a linear function of frequency in the θ band. This linear phase property can be easily explained by the evoked response model and therefore does not require more sophisticated nonlinear oscillator models.

Based on systems theory (Oppenheim et al., 1997), if the stimulus-response phase lag changes linearly across frequency, it indicates that the evoked response has a finite duration and has a symmetric waveform centered at the group delay (Fig. 1*A* for a delay system, for example). The current results suggest that the EEG response resembles the speech envelope but delayed, which supports the evoked response hypothesis. It is possible that cortical activity tracks the speech envelope or related features (Ding and Simon, 2014), and it is also possible that discrete acoustic landmarks that are extracted using nonlinear mechanisms drive the evoked responses (Doelling et al., 2014).

The group delay observed here is around 150 ms. Above 8 Hz, neural activity is not precisely synchronized to the speech envelope. Below 4 Hz, the phase linearity was weaker, suggesting more complex generation mechanisms. A previous MEG study finds similar group delay for the response to amplitude modulated tones: the group delay is 131 and 147 ms in the left and right hemispheres respectively, in the frequency range between 1.5 and 8 Hz (Wang et al., 2012). The 150- to 200-ms group delay also corresponds to the latency of the N1 and P2 responses in the temporal response function derived from the envelope tracking response (Aiken and Picton, 2008; Lalor et al., 2009; Ding and Simon, 2012a; Horton et al., 2013).

The current study finds that the stimulus-response phase lag changes approximately linearly across frequency (Figs. 2*B*, 3*A*), and participants who have higher stimulus-response phase coherence generally showed the better phase linearity (Fig. 3*B*). A previous MEG study, however, has shown that the stimulus-response phase lag cannot be explained by simple evoked responses (Doelling et al., 2019) but is more consistent with the prediction of a nonlinear oscillator model (illustrated in Fig. 1*B*). These two studies, however, focus on the neural responses in different frequency bands and during different tasks. The study by Doelling et al. (2019) analyzes cortical activity synchronized to auditory rhythms at 0.5, 0.7, 1, 1.5, 5, and 8 Hz. Four of the 6 frequencies considered in the study are below the θ band, and the current study also finds that the stimulus-response relationship is complicated below the θ band. Therefore, the results from these two studies do not conflict but reveal different neural mechanisms in different frequency ranges.

During speech processing, the neural response below the θ band can encode higher-level linguistic structures, e.g., phrases and sentences, on top of slow acoustic modulations, even if these linguistic structures are mentally constructed based on syntactic rules instead of prosodic information (Ding et al., 2016a). These results suggest that multiple factors could drive very low-frequency neural synchronization to speech. The analysis in this study characterizes neural synchronization to the speech envelope and cannot capture purely syntactic-driven response components. In other words, the neural response shown here is the response to acoustic modulations in speech, instead of the response to linguistic structures. The slow acoustic modulations below the θ band, however, could serve as a prosodic cue for mental construction of phrasal-level linguistic structures (Frazier et al., 2006). It is possible that distinct mechanisms are employed to encode syllabic-level and higher-level speech information: a roughly linear code is employed to encode syllabic-level speech features while more complex neural mechanisms are employed to prosodic features, which allows for frequent interactions with the syntactic and semantic processing systems.

In sum, by analyzing the stimulus-response phase lag, we show that the speech response in the θ band was approximately a delayed version of the speech envelope in the same frequency range. A time-delay system can be readily implemented using a linear time-invariant system, which is consistent with the evoked response hypothesis. Future studies, however, are needed to study whether the response phase property is modulated by attention and whether similar results could be obtained when listening to other sound.

## Synthesis

Reviewing Editor: Christine Portfors, Washington State University Decisions are customarily a result of the Reviewing Editor and the peer reviewers coming together and discussing their recommendations until a consensus is reached. When revisions are invited, a fact-based synthesis statement explaining their decision and outlining what is needed to prepare a revision will be listed below. The following reviewer(s) agreed to reveal their identity: Bernhard Ross, Alexander Billig.

The reviewers agree that the manuscript provides an important contribution for understanding cortical tracking mechanisms for speech. The methods and analyses are appropriate, although there are some technical details that could be better explained in the manuscript. The results are straight forward and well described. In general, improving readability of the manuscript and generalization of the results would be helpful.

Reviewer 1:

Several studies reported an association between the envelope of speech sound and low-frequency brain responses recorded with EEG or MEG while participants listened to ongoing speech. The significance of such observations and the underlying neural mechanisms are matter of current research. The authors investigated the phase relationship between the stimulus speech envelope and the EEG signal. Their hypothesis was that a linear system would be characterized by a constant time delay between sound envelope and EEG, equivalent to a linearly increasing phase difference with increasing frequency. In contrast, the case of a non-linear oscillatory system would predict a constant phase difference over a certain frequency region. Participants in the study listened to a total of two hours of ongoing speech. The main results was a significant phase locking between the speech envelope and the EEG signal in the 3 Hz to 8 Hz frequency range and a constant group delay within this frequency band. Based on these findings, the authors concluded a linear system underlying the generation of brain rhythms following the speech envelope rather than a system of non-linear oscillators.

The research question is relevant and timely. The authors developed a clearly defined hypothesis. They conducted an experiment that potentially could support the hypothesis. The experimental work and the data analysis was performed according to the state of art in the area of research. The manuscript is organized according to the requirements of the journal and is well written. The discussion is based on the authors findings.

The innovative highlight of the study is the analysis of very long EEG time series which allowed to obtain a high signal to noise ratio for the phase measures.

The study design, the description of the methods, and the report of the results are pretty straightforward. However, the manuscript contains redundancies and could be shortened substantially, which would improve readability and access to the manuscript.

There are a number of details, mostly of technical nature, which should be addressed to improve the manuscript. The details are outlined in order of appearance in the manuscript:

Details:

Pg 3, 2nd paragraph: The authors introduce the linear time-invariant (LTI) system and a reference to the temporal response function (TRF) frame work. The latter is not necessary. The work by Ding and Simon (2012) and Lalor et al. (2009) is about de-convolution techniques to estimate a TRF. However, this has not been applied in the current study. The authors should stay with the LTI system, especially because concepts like the group delay are defined within the LTI framework.

Pg 3: the references to Figure 1 and the related discussion should be moved from the introduction section to the results or discussion section.

Pg 6: The last paragraph of section 2.4 and section 2.5 could be combined to emphasize that both the EEG signal and the speech envelope signal were treated in the same way by low-pass filtering and down sampling.

Pg 6, sect. 2.6: remove the redundancy ‘the Discrete Fourier Transform (DFT) implemented using’

Pg 6, sect. 2.6: revise the sentence starting with ‘If’ to something like: The response and stimulus phases were denoted as αft and βft in the frequency bin f and time bin t, and the stimulus-response phase lag θft was calculated as αft − βft.

Pg 7: The definition of the phase coherence C(f) is not consistent with the reference to Peelle et al. (2013), who used a coherence measure which was bound to the interval [0.1]. However, C(f) increases with the number of samples in time. You may want to divide the right side of the equation by N. You refer to this issue in the last paragraph of section 2.6. However, the definition of C(f) is still not consistent with the critical value for the Rayleigh test (approx. 3.0 for p=0.05). Please see page 70 Eqn 4.18 in the book by Fisher (1993), cited in section 2.7.

Pg 7: A set of 14 fronto-central electrodes was selected ‘to characterize’ ... Please be more specific how the signals from multiple electrodes were combined. Specifically was the mean of the positive definite coherence measure calculated (which constitutes a bias) or was the mean calculated in the somplex domain?

Pg 8: Section 2.8 is an example for highly redundant writing. E.g., ‘The phase coherence was always positive’ ... not necessary because that is how C(f) was defined. The whole section could be reduced to a single sentence that the signed coherence was defined by multiplying C(f) with the sign of the cosine part. However, a topographic map of the signed coherence has not been shown in the results section. Thus, the section 2.8 should be eliminated.

Pg 9. section 2.9.1 describes a nonparametric permutation test for the phase coherence. However, Section 2.6 explains that the parametric Rayleigh test had been used. Thus, the nonparametric test is not necessary and should be removed.

Pg 9, section 2.10 The authors introduce the definition of the LTI system. Again, for better clarity, the authors should stay within the LTI frame work and remove the references to TRF.

Pg 10: A main concern about modelling a non-linear oscillatory system is that it is not clear what is different and new compared to the cited study by Doelling et al. (2019) (besides that their study is about music).

Pg 11 1st paragraph: grammar: ‘were averaged across all electrodes’. Was this an average across all electrodes or the selected 14 fronto-central electrodes?

Pg 11, 1st paragraph: Remove redundancies: If selection of 14 fronto-temporal electrodes was a planned analysis, describe this in the methods section, it it was data driven, describe it in the results section, but not both.

Pg 11 and Figure 2A: What is the meaning of the y-axis scale: Where is the significance threshold? For what alpha level?

Pg 11 and Figure 2: Figure 2B illustrates that the phase lag could not be measured reliably above about 17 Hz. Thus, the x-axis scale could be limited to 0-17 Hz (or less), which would improve the illustration of the main effects. There is no group statistics available. Commonly, these type of data show noticeable between participant variability. Some descriptive statistics could document such variability. Moreover, the graphs in 2A - 2D should be completed with confidence intervals for the group means.

Pg 11 3rd paragraph: Remove redundancies: The definition of the group delay does not need to be repeated here. Instead, provide statistical justification for the claim that the group delay was consistent across participants between 3.5 Hz and 8 Hz.

Pg 12 2nd paragraph: The description of the LTI system is very simplistic and repeats that the characteristics of a constant group delay had been already introduced. Alternatively the authors could discuss limitations of a strictly linear model. It could be that the evoked response is triggered by a time discrete nonlinear process. For example, preprocessing in the auditory system could lead to identify a series of discrete events (e.g. associated with envelope transitions) which in turn elicit series (of partly overlapping) transient evoked responses .

Reviewer 2:

The authors analyze the relationship between phase and frequency in cortical envelope-tracking of natural speech. They look to distinguish between entrainment and transient response superposition as accounts for envelope-tracking. They find a relatively linear relationship between phase and frequency between 3.5 and 8 Hz, suggesting that evoked responses may account for tracking in this frequency range during passive listening to speech

The method and analyses seem appropriate, the manuscript is concise and generally clear. I believe the paper makes a useful contribution in advancing understanding of circumstances under which different tracking mechanisms are dominant. I have a couple of questions about the strength and generality of the linearity result.

The authors make the reasonable definition of linearity as the reciprocal of the absolute value of the second derivative. Under this measure, a perfectly linear phase-frequency relationship gives an infinite value of linearity. The plot in 2B - which presumably reflects an average over the group of participants - indeed looks close to linear in the relevant range, and the statistical testing reveals more linearity than expected by chance. However, I question how reasonable it is to summarise this result as “a linear relationship”. 2C suggests the peak linearity is below 1.5, suggesting that the mean absolute second derivative is above 2/3, rather than zero. Would the authors have described the relationship as linear regardless of the value, so long as the relationship was more linear than expected by chance? Is a safer claim that there is “some linearity” in the relationship, and that evoked responses therefore make a non-zero contribution to tracking in this frequency range? Perhaps the authors can also directly compare linearity in this frequency range to that in the lower and higher frequency ranges, to also argue that the relationship is signifcantly more linear in the theta range than the sub- and super-theta ranges?

The clustering of the individual participant dots in the frequency range of 2D were striking and helpful to the reader. To further understand the strength of the linearity result, it could be helpful to see plots of the phase-frequency relationship in different individuals. How similar do they look to what is presumably the group plot in 2B?

Minor English comments

Pg 1 Sig statement should read “the delta-band envelope-tracking response” or “delta-band envelope tracking responses"

Pg 2 Intro “i.e. show” does not make sense

Pg 3 should read “computationally explicit"

Pg 3 should read “validity of using"

Pg 6 should read “The envelopes of the stimuli were"

Pg 11 should read “results in Fig. 2A were"

Pg 11 should read “Based on systems theory” (twice)

Pg 13 should read “are below 3.5 Hz"

Pg 13 should read “but reveal"

Pg 13 should read “The analysis in this study characterizes"

Fig 1 “Since the phase lag ... “ sentence doesn’t make sense on its own alone
